# Addressing noise in co-expression network construction

**DOI:** 10.1093/bib/bbab495

**Published:** 2021-11-30

**Authors:** Joshua J R Burns, Benjamin T Shealy, Mitchell S Greer, John A Hadish, Matthew T McGowan, Tyler Biggs, Melissa C Smith, F Alex Feltus, Stephen P Ficklin

**Affiliations:** Department of Horticulture, 149 Johnson Hall. Washington State University, Pullman, WA 99164. USA; Department of Electrical & Computer Engineering, 105 Riggs Hall. Clemson University, Clemson, SC 29631. USA; School of Electrical Engineering and Computer Science, EME 102. Washington State University, Pullman, WA 99164. USA; Molecular Plant Sciences Program, French Ad 324g. Washington State University, Pullman, WA 99164. USA; Molecular Plant Sciences Program, French Ad 324g. Washington State University, Pullman, WA 99164. USA; Department of Horticulture, 149 Johnson Hall. Washington State University, Pullman, WA 99164. USA; Department of Electrical & Computer Engineering, 105 Riggs Hall. Clemson University, Clemson, SC 29631. USA; Department of Genetics and Biochemistry, 130 McGinty Court. Clemson University, Clemson, SC 29634. USA; Biomedical Data Science & Informatics Program, 100 McAdams Hall. Clemson University, Clemson, SC 29634. USA; Clemson Center for Human Genetics, 114 Gregor Mendel Circle, Greenwood, SC 29646. USA; Department of Horticulture, 149 Johnson Hall. Washington State University, Pullman, WA 99164. USA; School of Electrical Engineering and Computer Science, EME 102. Washington State University, Pullman, WA 99164. USA

**Keywords:** co-expression, networks, noise, multidimensional, gene expression

## Abstract

Gene co-expression networks (GCNs) provide multiple benefits to molecular research including hypothesis generation and biomarker discovery. Transcriptome profiles serve as input for GCN construction and are derived from increasingly larger studies with samples across multiple experimental conditions, treatments, time points, genotypes, etc. Such experiments with larger numbers of variables confound discovery of true network edges, exclude edges and inhibit discovery of context (or condition) specific network edges. To demonstrate this problem, a 475-sample dataset is used to show that up to 97% of GCN edges can be misleading because correlations are false or incorrect. False and incorrect correlations can occur when tests are applied without ensuring assumptions are met, and pairwise gene expression may not meet test assumptions if the expression of at least one gene in the pairwise comparison is a function of multiple confounding variables. The ‘one-size-fits-all’ approach to GCN construction is therefore problematic for large, multivariable datasets. Recently, the Knowledge Independent Network Construction toolkit has been used in multiple studies to provide a dynamic approach to GCN construction that ensures statistical tests meet assumptions and confounding variables are addressed. Additionally, it can associate experimental context for each edge of the network resulting in context-specific GCNs (csGCNs). To help researchers recognize such challenges in GCN construction, and the creation of csGCNs, we provide a review of the workflow.

## Introduction

The first form of a gene co-expression network (GCN) (also called a relevance network) was reported in 1998 [1]. Since then, GCNs have been used for a variety of transcriptomic analyses across a range of living organisms to provide clues to the context in which genes interact to identify novel gene candidates coordinating specific biological functions, to identify genes underlying complex traits of interest (i.e. systems genetics analyses) [2, 3], to translate knowledge about gene activity between species [4, 5], to study evolutionary changes in gene modules [6, 7], to improve identification of significant markers in Genome-Wide Association Studies [8–10] and as prior knowledge in regulatory network construction [11].

A variety of software tools have been developed that use either microarray or RNA-seq data to assist researchers to create GCNs. These include WGCNA [12–14], CLR [15], MRNET [16], RMTGeneNet [17], petal [18], INfORM [19] and FastGCN [20]. Because the construction of GCNs involves pairwise calculations, the algorithm is highly parallel, and developers of some of these software have introduced modules for accelerated computing on graphical processing units (GPUs) [20]. When constructing a GCN, a similarity test such as Spearman, Pearson, Kendall Tau correlation, biweight midcorrelation [21] or mutual information (MI) [16] is applied to each pairwise comparison. After similarity testing, values below a given threshold are excluded from the network. This threshold is determined using *ad hoc* methods [22, 23], permutation testing [24], linear regression [24], spectral graph theory [24], random matrix theory (RMT) [25], topological properties [26], Fisher’s test of Homogeneity [27], supervised machine learning [27, 28] or rank-based methods [29, 30].

Typically, significance thresholds are set at relatively high stringency levels to ensure that network properties are preserved, and false relationships are limited. Unfortunately, such high correlation thresholds are indicative of high levels of noise. For example, the RMT approach is a thresholding method that identifies a correlation value below which the GCN begins to exhibit properties of a random network (i.e. too much noise). It is our experience that RMT identifies the point at which GCNs begin to exhibit random properties typically between ±0.85 and ±0.95 correlation. Such high correlation thresholds exclude moderate relationships (e.g. ≥ ±0.5) that are obfuscated by high levels of noise. This is disappointing for researchers who may fail to find the meaningful relationships that exist below the threshold.

There are multiple sources of noise in gene expression data that may result in high network thresholds. Noise can result from natural intrinsic variation (stochastic differences within a cell), extrinsic variation (differences between homogenous cells) [31–33] and heterogeneity caused by multiple experimental variables (e.g. genotype, treatment, developmental stage, tissue, etc.). Non-natural sources include systematic noise from variation in data collection and measurement and statistical bias where methods may be applied inappropriately.

Accounting for intrinsic and extrinsic noise is important with single-cell expression data and is an active area of research [34, 35]. Here, we focus on RNA-seq data where samples include mixed cell types, and intrinsic and extrinsic noise cannot be accounted for, but heterogeneity caused by experiments with multiple variables can be accounted for. Single experiments may include large numbers of samples across multiple conditions, or sometimes researchers seek to combine public datasets from large public repositories such as NCBI Gene Expression Omnibus [36] and the Sequence Read Archive (SRA) [37], which may have multiple experimental conditions. In both cases, as datasets become larger and more diverse, derived GCNs become less informative due to an increase in noise from multidimensionality [38].

One way to improve the utility of a network is to address the causes of noise. Two common approaches used to address noise in GCN construction are downsampling and aggregation. Downsampling subdivides samples either by manually grouping them via experimental conditions [39], or automating grouping by methods such as *k*-means clustering or randomization [40, 41]. Downsampling is employed before network construction to capture relationships that may be context-specific. Aggregation is performed after network construction and forms a consensus across multiple networks by ranking edges that are conserved [42, 43]. Aggregation is meant to reduce noise by lowering the rank of edges with limited reoccurrence. Hybrid approaches have also been employed to capture the benefits of both methods [38, 44].

One major reason for downsampling is that researchers often seek to identify context-specific relationships between genes. In the literature, a context-specific network is referred to as condition-specific, context-specific, tissue-specific, trait-related, condition-dependent or targeted networks. We refer to networks of such relationships as context-specific GCNs (csGCNs). For example, a subgraph, where all edges in the network consist of relationships that are associated with a specific tissue, treatment, phenotype, etc., is context-specific. A researcher may perform downsampling, for example, by separating samples into a group measuring a treatment and another for control, and creating a separate treatment GCN for each. Unfortunately, the use of downsampling to generate csGCNs is increasingly challenging as the dimensionality of experiments increases. For example, how is one to divide samples into groups when gene expression is a result of multiple conditions at one time (e.g. genotype, treatment and time point) without sacrificing statistical power? Also, downsampling cannot resolve noise from confounding variables (which we demonstrate later). Additionally, the aggregation of networks seems biased toward contexts that are more prevalent across the experiments and edges that are specific to a single context would be more likely not to be present in the network.

**Figure 1:**
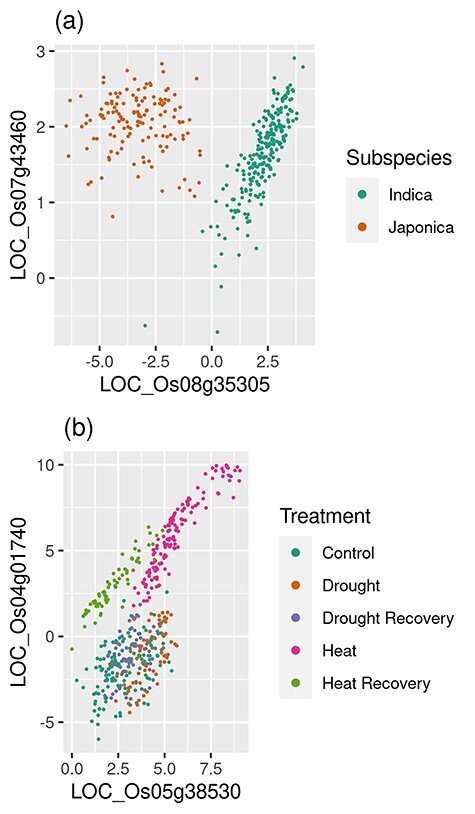
Examples of pairwise condition-specific gene co-expression. RNA-seq expression data were from the NCBI SRA Project PRJNA301554. The figure includes scatter plots of gene pairs with condition-specific co-expression for (**A**) two rice subspecies and (**B**) different experimental treatments.

Neither downsampling nor aggregation fully addresses issues of noise. Consider the two scatterplots of Figure 1. These plots show pairwise gene expression from NCBI’s SRA [37] PRJNA301554 project [45], which consists of gene expression from four genotypes of *Oryza sativa* (rice) that were exposed to drought, drought recovery, heat, heat recovery and control regiments measured every 15 min over several hours. This experiment was selected because it includes multidimensional data from a controlled experiment with different treatments, genotypes (and subspecies) and time points.

Figure 1**A** shows a gene pair with two distinct modes (clusters) of co-expression for each rice subspecies. The application of correlation considers all samples, and this pair results in a Spearman’s *rho* of −0.003. Such a low score would exclude this pair from a GCN. If only Indica samples were included (such as if samples were downsampled by subspecies), then a Spearman’s *rho* of 0.82 would result and the Japonica samples would show little or no correlation.

In Figure 1**B**, co-expression only exists distinctly for heat and heat recovery conditions but very little for the others. Overall, this pair exhibits a Spearman’s *rho* of 0.62, which is often too low for most thresholding approaches, and the edge is lost despite that heat and heat recovery samples together exhibit a correlation of 0.98. However, suppose this pair were to be included in the network at the 0.62 correlation value. The edge would be accurate because the gene pair is correlated in heat, but the context that the pair is only co-expressed in heat is lost.

In both plots of Figure 1, the problem of systematic noise is also demonstrated. Students of introductory statistics are taught to check that assumptions of a test are met before using that test. Yet for GCN construction, checks of correlation assumptions are often overlooked—most likely due to the high computational demand for checking every pairwise comparison. Pearson is a commonly used correlation method in GCN construction and assumes no outliers, equal variance and linearity. These assumptions are not met in the examples of Figure 1—therefore, the strength of the relationship is suspect. In other cases, inappropriate application of correlation can lead to erroneous inclusion or exclusion of edges when applying a threshold. Spearman seems a better choice as it only assumes monotonically increasing values, but it should not be applied with multi-modal data such as the visible clusters of Figure 1, and again, the conditional context and strength of the relationship of Figure 1**B** would be lost with Spearman.

The plots of Figure 1 were specifically selected to demonstrate the issues of noise and statistical bias; however, to quantify the potential statistical bias, we generated a traditional GCN using WGCNA from the PRJNA301554 dataset and tested the best 15K edges. After testing both for normality (Royston test; α = 0.01) and equal variance (Breush Pagan test for the presence of heteroscedasticity; α = 0.01), 97.3% of the edges in the network did not meet one or both assumptions even after outliers were removed. Thus, Pearson is largely unsuited for this dataset. WGCNA uses the biweight midcorrelation test rather than Pearson, yet it too is a linear model with similar assumptions. Of the edges that exhibited non-normal co-expression, 75% were multi-modal like Figure 1**A** [identified using Gaussian Mixture Models (GMMs)]. Thus, Spearman is largely unsuited for most of the data as well. Also, the RMT approach identified a correlation threshold of ±0.91—an extremely high correlation threshold indicating high levels of noise. Again, RMT thresholds indicate the point at which the network begins to exhibit random properties. These results imply that most of the edges in the network are biased.

We should note that not all datasets exhibit such a high level of bias. For example, the WGCNA package uses for its tutorial a dataset containing 3600 genes from a compendium of mouse liver microarray measurements [46]. When the same tests of normality and equal variance were applied to the GCN constructed from that data, 92.6% of the edges met both assumptions—a highly contrasting result to the rice dataset. Therefore, GCNs constructed from this dataset should have low statistical bias. The RMT threshold for the mouse liver dataset is 0.81. Although much lower than the rice data, it is relatively high indicating there may be some noise adding too much variability. Relationships below 0.81 that may be meaningful are not explored due to this noise. The rice and mouse liver datasets demonstrate that gene expression data can vary widely in terms of bias and researchers should be aware of such bias as it affects the GCN construction approach they may have chosen to use.

Here, we describe a workflow that will allow researchers to account for both the natural noise of large multivariable datasets and statistical bias that negatively affect GCN construction. The workflow is not new, as it uses the Knowledge Independent Network Construction (KINC) software [47] and has been used in several recent plant, human and data management studies. These include identification of csGCNs that are specific to tumor types in human cancer [48, 49], normal brain tissue [50], post-harvest-specific networks in domesticated apples (*Malus domestica*) [51], root nodulation biomarkers in *Medicago* [52], ripening in d’Anjou pears [53] and exploration of the effects of ‘lossy compression’ of GEMs [54]. Here, we describe sources of noise, compile a review of the workflow and describe the computational performances and limitations. The objective is to help researchers consider sources of noise in their increasingly multidimensional transcriptomics datasets used for GCN construction, to apply available solutions and to foster further advances.

## Noise reduction strategy

Given high levels of potential noise in gene expression datasets, we suggest that the ‘one-size-fits all’ approach of most network construction tools is not appropriate for all datasets. The examples of Figure 1 indicate that gene co-expression can be unique at each pairwise comparison. Therefore, each gene pair should be tested separately. This contrasts with downsampling, which divides samples into groups and creates GCNs separately for each group but applies the same statistical methods to all comparisons. By performing tests on each pair separately, the unique patterns of expression between genes can be accounted for. Testing each pair separately requires more computational power—which we provide details for below—however, it has the advantage of addressing sources of noise specific to each unique gene pair and can identify the context (or conditional association) for each pair as well.

**Figure 2:**
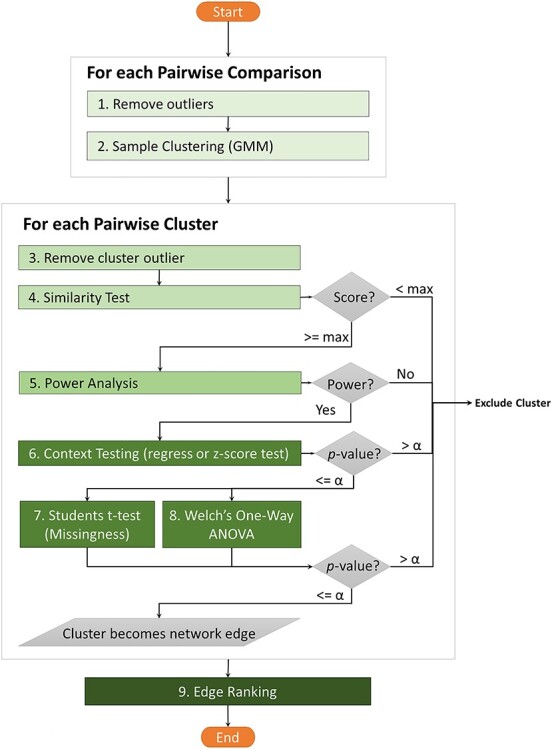
The KINC GCN construction process. The flowchart depicts the eight steps of the KINC workflow for addressing statistical and natural noise in GCN construction. In summary, each pair of genes proceeds through the workflow. First, outliers are removed. Second GMM is performed to identify clusters of expression. Third, cluster outliers are removed and fourth the similarity test (e.g. Pearson or Spearman) is performed. Clusters with a minimum score proceed. Fifth, a power analysis is performed to ensure sufficient statistical power in the correlation test. Clusters with high score proceed. Sixth, clusters are tested for association with context (e.g. experimental conditions) and those with significant *P* values are associated with the condition and proceed. Seventh, parallel tests for similar patterns of missingness (*t*-test) and difference in variance (Welch’s one-way ANOVA) are performed. Clusters with significant *P* values are retained as context-specific edges in the network. Finally, all edges are ranked according to *P* values and scores to help researcher prioritize edges.

The following is a step-by-step description of the KINC csGCN construction workflow that addresses noise at the pairwise level. Most steps are performed at the gene–gene pairwise comparison level. A flowchart of this workflow is presented in Figure 2.

### Preprocessing the GEM

Before execution of the following workflow, some preprocessing of the input Gene Expression Matrix (GEM) may be required. For researchers who combine samples across experiments, it may be necessary to remove batch effects using tools such as ComBat [55], ComBat-seq [56], SVA [57] or SVAseq [58]. Systematic noise resulting from different approaches to sample library construction may result in bias that limits any GCN software, including KINC. Also, before analyzing RNA-seq data for both DEG analysis and GCN construction, it is common practice to filter genes in samples with low counts. EdgeR recommends filtering genes that do not meet a set counts per million threshold in a minimum number of samples [59]. DESeq2 expands on this by using a mean normalized count [60]. Such forms of filtering can occur, at the discretion of the researcher, before using the workflow. Such types of adjustments are not provided by KINC as existing tools adequately perform these tasks.

#### Step 1. Outlier removal

For each pair of genes, the first step is outlier removal. Outliers can bias the clustering step that follows and should be removed. Outliers are identified from all the pairwise sets of values using the Tukey method.

#### Step 2. Pairwise sample clustering

To address natural noise from experimental variation, GMM clustering is applied at each pairwise gene comparison (before correlation). This step addresses noise separately for each gene pair. Stated simply, GMM detection is an unsupervised, clustering approach that fits one or more potentially overlapping Gaussian density distributions over the pairwise data. An in-depth description of GMMs as used by KINC is provided by Shealy and Burns, *et al.* [47]. Users of KINC can specify the minimum allowed size of a cluster. There are other clustering approaches available but currently, KINC only supports GMMs. Execution of GMMs is computationally expensive and KINC uses a GPU implementation to improve performance.

#### Step 3. Cluster outlier removal

The second round of outlier removal is performed on each cluster identified by the previous step. Outliers are removed because the subsequent similarity step may be biased by outliers. For example, Pearson correlation requires that no outliers exist in the data. Spearman is more robust in the presence of outliers but removing them will not prejudice the tests. Also, because clusters follow a Gaussian distribution, they are certain to meet other assumptions of both Spearman and Pearson tests.

#### Step 4. Similarity scoring

For each cluster, the similarity score is measured. For pairs with multiple clusters, similarity will be calculated multiple times, once for each cluster. For large gene sets, billions of scores will be calculated and the resulting output file may be extremely large. Therefore, KINC users can set a minimum correlation value to limit output size. By default, KINC sets this to ±0.5 to allow for meaningful correlations while reducing storage requirements. Clusters that do not meet the minimum score are excluded. This filter is simply to limit output file size. If sufficient storage space is available, then all correlation values, for all pairwise tests, will be passed to the later steps for filtering.

KINC supports both Pearson and Spearman correlation methods. Regarding MI, studies have shown that, in general, MI performs no better or worse than correlation methods, Lindlöf and Lubovac indicated they did not detect any difference between GCNs constructed via correlation versus MI approaches. However, they attributed that to a possible bias toward linear relationships in their data due to the sampling approach [61]. Song *et al*. tested their biweight midcorrelation approach against several other methods, including MI and concluded, using both real and simulated data that MI tends to be inferior and suggested a spline-based regression model as an alternative to MI approaches. They note that MI is more meaningful when sample sizes are larger (e.g. *n* > 300 samples) [21]. Lastly, Huang *et al*. attempted to optimize GCN construction by comparing multiple techniques as well. They also concluded that correlation-based approaches perform better for specific types of genes or specific types of interactions but that larger samples size were important for MI to perform well [62]. Consequently, KINC does not support MI.

#### Step 5. Power analysis

Next, a power analysis test is performed to remove clusters that have too few samples to justify the correlation score calculated in Step 4. For example, consider a dataset with 100 samples. Suppose that gene X is only expressed in five of those samples—perhaps it has condition-specific expression. For all pairwise comparisons of gene X with every other gene, only the five samples can be used for correlation as all other samples must be excluded as they have no expression for gene X. Suppose in those five samples the correlation of gene X and a gene Y is 0.85. A power analysis calculation would indicate that for a Type I error rate of 0.001 and a Type II error rate of 0.20, we would need at least 14 samples to determine if a correlation value of 0.85 differs from zero. Therefore, such a test would be underpowered with only five samples and should be excluded. Underpowered correlation tests occur when there are too many missing values for a gene or too few samples in a cluster identified by GMMs. The former case is an unaddressed problem for all GCN construction tools, and the latter is specific for this approach. By default, KINC requires a power of 0.8 (1 minus the type II error rate of 0.2) with a significance value, α, of 0.001. Clusters with insufficient power (i.e. have too few samples for the correlation level) are excluded by KINC. Users can set different power and α limits.

#### Step 6. Context testing

To add context, each cluster that passes the power analysis test is tested for association with experimental variables (e.g. treatment, genotype, developmental stage, tissue, environment, etc.) and *P* values, one per variable, are assigned to each cluster. For categorical variables, clusters undergo two z-score tests of proportions. The first tests enrichment of the category within the cluster and the second tests that the category tends to not appear outside of the cluster. By default, clusters with a *P* value <0.001 in both tests are retained. For quantitative variables (such as a time series or clinical measurement), linear regression is used and by default, those with an *R*^2^ > 0.3 and *P* value <0.001 are retained.

#### Step 7. Correct bias in context associations: missing values

A gene has missing expression in a sample if there are no counts for it from the RNA-seq data, or if it was removed through a low-count filter before network construction. This does not imply the gene was not expressed, but that it was not detected. During clustering (Step 2), samples with missing values, in either one of the genes, are excluded because the two genes cannot be compared when one sample has a missing value. The pattern of missing values within each gene may have a biological source. For example, suppose gene X and gene Y have count values only in one treatment, say a control treatment and in no other treatment. Step 6 will associate their cluster with the control treatment because the only samples in the comparison are from the control treatment. Their missing patterns should be similar. Next, consider that a gene Z has basal function, has count values in all treatments, and tends to correlate with many genes, perhaps due to Circadian control in a time-series experiment. When gene X and Z are compared, Step 6 will also associate this pair with ‘control’ because the only samples in the comparison are from control. This is despite that gene Z has ubiquitous expression and is not ‘control’ specific. Any gene that correlates with X will always be associated with control resulting in a ‘spoke’ like appearance of connected genes around X in the context-specific network. To reduce such spokes, where context-only genes correlate with condition agnostic genes simply due to patterns of missing values, a Student’s *t*-test is used to ensure that two genes have similar patterns of missingness (*P* value <0.001). Thus, genes X and Y in the example would be considered context-specific for control but gene X and Z would not. We acknowledge that this approach may be overly conservative for all comparisons and more work is needed to improve such filtering.

#### Step 8. Correct bias in context associations: confounding variables

**Figure 3:**
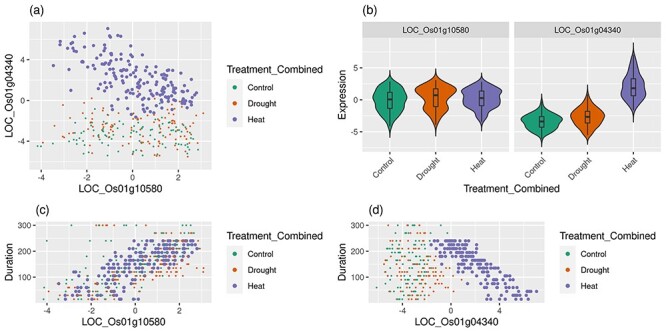
Confounding variables in gene co-expression: Heat example. The expression scatterplot of a rice gene pair is shown. The pair in (**A**) is poorly correlated overall (SCC = −0.13) but moderately correlated if only the heat samples are considered (SCC = −0.63). In (**B**) only the LOC_OS01g04340 gene has a visible difference in expression in the heat response with the LOC_OS01g04340 gene showing a visible increase in expression in heat samples. This results in the purple cluster of genes distinctly separated from other samples in (**A**). In (**C**) and (**D**) both genes exhibit a linear relationship with time but LOC_Osg04340 only exhibits time-dependence in heat samples. This covariance of both heat and time in LOC_OSg04340 falsely result in this pair being associated with heat when it is only correlated by time in heat.

Another reason for the incorrect association during Step 6 is from multivariate control of gene expression. Step 6 association is meant to identify when a cluster is associated with a single variable in the data. This variable could be a category (e.g. heat treatment) or quantitative (e.g. time). However, consider the case where the expression of gene X is a function of two variables such as heat response and time, and a gene Y’s expression is a function of circadian control, and not heat. Figure 3 shows such an example. Figure 3**A** shows the pairwise scatterplot of two genes with samples colored by treatment. The purple heat samples would be identified as a cluster in Step 2 with moderate inverse correlation [Spearman Correlation Coefficient (SCC) = −0.63]. Step 6 would then associate the heat treatment with the cluster. Observationally, there does appear to be a heat-specific cluster in Figure 3**A**. Yet, Figure 3**B** shows that heat samples have a different mean expression in the LOC_Os01g04340 gene indicating a heat-specific response in that gene but not an obvious heat response in the other. Figure 3**C** indicates that the expression of LOC_Os01g10580 increases with time for all treatments, and Figure 3**D** indicates that the expression of LOC_Os01g04340 decreases with time, but only for the heat-treated samples. The expression of the first gene appears to only be a function of one variable—time (perhaps under circadian control). The expression of the second gene is a function of two variables—heat treatment and time (perhaps a waning response to heat treatment). Time is a shared variable for both genes, whereas heat only affects one gene. Because Step 6 is meant to identify relationships that are specific to only a single variable, this relationship (confounded both by time and heat) is a false association to heat alone. KINC excludes such false associations by performing a Welch’s one-way analysis of variance (ANOVA) test on each gene comparing the variance of the ‘in’ group with that of the ‘out’ group. The ‘in’ group consists of those samples that are in the cluster. The ‘out’ group could be samples in a specific category, such as ‘control’, or all other samples not in the cluster. This test ensures that the category being tested exhibits differential expression in both genes. Both genes must have a significant *P* value (α < 0.001) for the cluster to be retained. We acknowledge that this filtering is a bit harsh because, as in the case of Figure 3, the relationship is excluded for heat-specific and for time-specific, when it is time-specific. Thus, some relationships will be missed. Better approaches are needed. Of note, a downsampling approach would not correct this bias.

#### Step 9. Ranking edges

Only clusters that pass correlation test assumptions, have sufficient power, have significant *P* values for context association, have significant *P* values for the missingness test and have no confounding bias from other variables are retained. These clusters become edges in the csGCN. The network is a csGCN because each edge is annotated with a specific experimental variable, indicating the context in which that edge is expressed. A gene pair may have multiple edges (each for a different context), but each would have a different *P* value and/or R^2^ for the respective condition. Unfortunately, because a pair of genes can have multiple edges, methods such as RMT cannot be used for thresholding as the similarity matrix becomes multidimensional. Therefore, to help researchers prioritize edges, they are ranked using a valuation approach that includes the similarity score, *P* values from all tests and the *R^2^* value if linear regression was performed (for quantitative variables). The ranking provides an alternative to the traditional correlation similarity score to prioritize the best edges in the network. This ranking can help prioritize edges in very large csGCNs.

#### Step 10. Visualization

**Figure 4:**
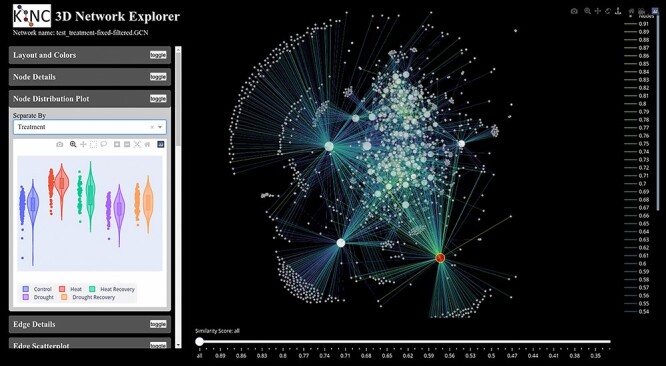
KINC GCN visualization. KINC provides a web-based tool for network visualization that allows the researcher to layer and color edges by their similarity score, *R*^2^ value, *P* values, rank, variable categories and relationship direction (negative or positive). The left sidebar provides useful plots such as scatter plots for selected edges, violin plots of expression for selected nodes, scale-free and clustering plots for the network and functional details about nodes.

Once a csGCN is complete, exploration of the network can occur. Users can filter the ranked edges, select experimental variables of interest or import the entire csGCN into the popular network visualization tool, Cytoscape [63]. Alternatively, KINC provides a 3-dimensional network viewer that allows the end-user to explore the csGCN by layering the edges by experimental variable, *P* values, similarity scores, etc. A screenshot of this tool is shown in Figure 4.

KINC v3.7 performs these steps and was written in the C++, OpenCL, CUDA, R and Python languages. It uses the Accelerated Computing Engine library v3.2.0 [64], which provides a mechanism for managing computational tasks on heterogeneous computational infrastructure. KINC is an open-source software package with source code available at the GitHub repository, https://github.com/SystemsGenetics/KINC and full step-by-step documentation at https://kinc.readthedocs.io/. The accompanying R package, KINC.R, is available at https://github.com/SystemsGenetics/KINC.R.

## Computational performance

The space and time requirements of the described workflow are most affected by the GMM calculations. Therefore, to demonstrate the computational performance of Steps 1–4, we use four different size datasets. The first dataset is a small yeast dataset with 7050 genes and 188 randomly selected RNA-seq samples from the NCBI SRA. The second is a medium-size dataset and is the same 475-sample rice dataset (PRJNA301554) described previously. The third is a subset of the rice dataset that contains only 141 samples that underwent drought stress. The fourth is a large GTEx [65] normal human brain tissue dataset consisting of the expression pattern of 56 202 genes across 1671 samples and 13 brain tissues [50]. The GTEx GEM was preprocessed by log2 transformation of the expression values obtained from GTEx, applying the Kolmogorov–Smirnov test to remove outlier samples and quantile normalization on the GEM. These datasets were selected to demonstrate computational performance for small, medium and large GEM sizes with different numbers of genes and samples.

To quantify computational requirements, we tested performance via KINC version 3.4.2 using GMMs on both the WSU Kamiak cluster and the Clemson University Palmetto cluster. Five compute nodes were used in parallel on Kamiak and each node provides two NVIDIA Tesla K80 (four GPUs each), 24 Intel Xeon E5-2670 CPUs and 256GB of RAM. The nodes that were used on Palmetto were equipped with Intel Xeon E5-2680 CPUs, 2 NVIDIA P100 GPUs and 128GB of RAM. Multiple iterations of testing occurred using between 16 and 120 CPUs and 1 and 17 GPUs in parallel.

KINC uses a binary encoding for storing the similarity matrix and GMM results. These output files were named with the extension ‘CCM’ (for the cluster correlation matrix) and ‘CMX’ (for the correlation matrix). The similarity matrix can become quite large {size of [*n* × (*n* − 1)]/2, where *n* is the number of genes}. KINC therefore automatically sets a default correlation threshold of 0.5 and uses a sparse matrix format to keep data files relatively small although end-users can change the cutoff as needed. We used this 0.5 cutoff for performance testing. The amount of storage space required for this experiment was also recorded.

**Figure 5:**
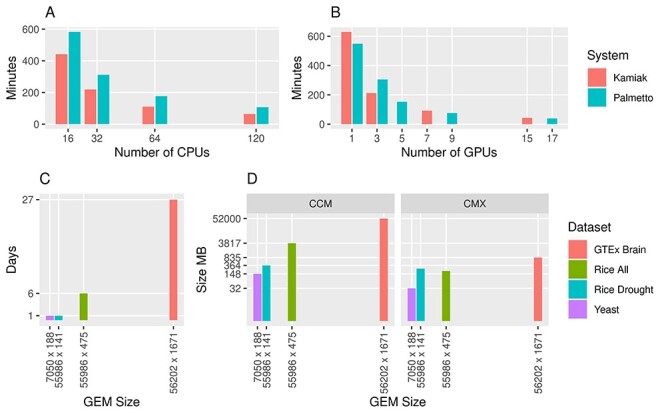
Computational performance of Steps 1–4 using KINC. Plots (**A**) and (**B**) indicate time of execution on a yeast (*Saccharomyces cerevisiae*) GEM containing 7050 gene transcripts and 188 samples on both CPUs and GPUs respectively. Performance measurements were measured on Clemson’s Palmetto HPC cluster and WSU’s Kamiak HPC cluster. Plot (**C**) indicates the time required to analyze GEMs of different dimensions on WSU’s Kamiak cluster using three GPUs. Plot (**D**) indicates the size in MB for the CCM file and the CMX file. KINC was instructed to only retain correlations whose absolute value was greater than or equal to 0.5. The GEM size axis in plots (**C**) and (**D**) is represented as the number of gene transcripts versus the number of samples.

Results show that the use of GMMs can be computationally time-consuming depending on the size of the GEM and large GEMs require larger amounts of storage. KINC can use both CPUs and GPUs concurrently across multiple compute nodes in parallel; This was fully tested using four different GEMs varying in size in both genes and samples from small (7050 genes × 188 samples) to large (56 202 genes × 1671 samples) (Figure 5). For the small yeast dataset (shown in Figure 5**A** and **B**), execution on Kamiak with 16 CPUs required several hours and execution on just a single GPU provided similar performance. The time was dramatically decreased as the number of CPUs and GPUs increased but with diminishing speedup. Even the dataset with more genes and similar samples (55 986 genes × 141 samples) was completed in less than a day with three GPUs (Figure 5**C**). Thus, small datasets can complete in a few hours to a day using similar computational resources that are increasingly available via institutional, national or cloud computing facilities. In contrast, very large datasets with thousands of samples can take weeks. Such large compute times may be impractical for some users. Additional research is needed to find methods that can reduce very large GEMs without loss of meaningful relationships or use other dimensionality reduction methods that are less computationally intensive.

## Limitations and areas to explore

The workflow described here is provided as a protocol that can be used to address issues of noise in GCN construction. One objective for this manuscript is to alert researchers to such issues and to foster the development of better tools, including those that can improve on this protocol. Here, we describe a few limitations. First, GMMs are not perfect in identifying all clusters. Because it is computationally intractable to explore all possible solutions, GMMs require random start locations, which may settle in different local minimums with different runs. We have observed improper identification of clusters when few samples are present or for genes biased by very low expression levels. The power-analysis step should filter clusters with few samples and context association testing will overlook clusters with no discoverable context. However, a more thorough examination of false edges resulting from the imperfect use of GMMs is needed.

GMMs may also suffer when context expression of genes overlaps in the 2D space. For example, consider the scatterplot of Figure 3**A**. The drought and control labeled samples overlap and are not distinguishable from one another. This is not problematic because there is no correlation within this group. However, if there were correlation in one treatment but not the other then KINC would fail to identify the cluster as context-specific. We believe the likelihood of overlap increases as more experimental variables are represented in the data. More work is needed to explore amelioration strategies to reduce the loss of sensitivity as more variables are included. The problem, however, limits the discovery of edges rather than producing false edges.

An additional challenge is that running this workflow on large GEMs may be difficult for some users who do not have access to GPUs or large compute clusters. We anticipate that as such resources become more widely available via institutional, national and commercial cloud computing, researchers will have access to these facilities. Given that facilities such as XSEDE [66], the Open Science Grid [67] and the Pacific Research Platform [68] are available to many researchers, more time-consuming analytical approaches can be used. Despite the computational challenges for analyzing multidimensional data, the simplistic traditional GCN methods are not adequate to account for bias at the pairwise level and researchers should attempt to account for them.

One topic absent from this manuscript is the measurement of the biological performance of csGCNs. Biological performance can be defined in terms of the number of true relationships that are represented in the csGCN and the lack of false associations. This is a challenging question to address for GCNs as well as csGCNs because of the lack of a gold standard, validated network by which GCNs can be compared, especially in all contexts. One of the most popular methods for measuring the performance of a GCN is comparing the number of conserved functional terms between neighbors in the network. This approach relies on the GBA concept that interconnected genes should share similar function. Extending ‘Guilt-by-Association’ by Degree [69] is one tool that uses a machine learning approach to measure how well a neighborhood of connected genes can predict the function of its connected neighbors. It has been shown, however, that these evaluation approaches may be biased toward genes that are more highly researched (i.e. have more annotated terms), multifunctional and with higher interconnections (or degree) in the network [70]. Another study shows that downsampling and aggregation improve the functional performance of GCNs [38]. Therefore, it seems reasonable to assume that downsampled and aggregated networks may be biased toward multifunctional genes. In contrast, csGCNs are context-specific and our assumption is they will be enriched for genes that are not constitutively expressed, have fewer annotations and tend to be less multifunctional. This would result in csGCNs performing worse in GBA studies and perhaps falsely imply that they perform poorly. This hypothesis should be explored further.

Despite the lack of a metric on biological performance, we assume that biological performance is improved in the csGCNs simply by ensuring that statistical biases are handled, and noise is accounted for using simple, commonly used statistical practices and methods. By ensuring that statistical assumptions are met, that tests have sufficient power and bias from missing values and confounding variables are accounted for, we conclude that the number of false edges should be reduced when exploring deeper into the correlation space that RMT would normally exclude.

Finally, the use of GMMs provides thousands of new context-specific edges that need exploration. KINC-derived networks can retrieve context-specific, statistically significant edges at correlation values as low as ±0.3 provided sufficient data (statistical power) are available. The biological role of correlation at such lowly correlated relationships is unknown. Are these primarily indirect relationships? More research is needed to determine the role of such significant but lowly correlated relationships.

## Conclusion

GCN construction is a widely applied technique that warrants improvement. As described here, multidimensional transcript profiles create challenges for traditional GCN construction due to multiple sources of noise and bias that are unaccounted for in traditional approaches. As previously noted, approximately 97% of edges in the rice dataset did not meet test assumptions. This result implies that the quality of traditional approaches (including the highly popular WGCNA) is highly dependent on the structure of the data. Thus, when researchers fail to find modules of interest it may be due to deficiencies of current GCN construction methods for multidimensional data rather than a lack of ‘signal’ in the data.

## Data availability

No new data were generated or analyzed in support of this research.

**Key Points**

- Multidimensional gene expression data contain natural and systematic noise that affects GCN results.
- The ‘one-size-fits-all’ approach to co-expression network construction cannot correct for noise.
- Sources of noise are different for each pairwise comparison so correction strategies should be applied at the pairwise level.
- The KINC toolkit offers an approach for pairwise correction of bias.

## Notes

**Joshua J.R. Burns** is a computer engineer and research associate at the Department of Horticulture at WSU with expertise in parallel and graphical processing unit programming.

**Benjamin T. Shealy** is a PhD candidate at the Department of Electrical and Computer Engineering at Clemson University. His current research focuses on AI-powered support tools for data-intensive scientific workflows.

**Mitchell S. Greer** is an Electrical Engineering and Computer Science BS graduate from WSU who now works in industry.

**John A. Hadish** is a graduate student in Molecular Plant Science at Washington State University. His research is currently concentrated in transcriptomic bioinformatics.

**Matthew T. McGowan** is pursuing a PhD degree in molecular plant science at WSU. His research interests include bioinformatics, statistical genomics and multi-omic data integration.

**Tyler Biggs** is a postdoctoral researcher at the Department of Horticulture at WSU focusing on the application of machine learning methods for feature discovery in large molecular datasets.

**Melissa C. Smith** is a professor in Computer Engineering at Clemson University and is focused on the use of emerging computing architectures and machine learning for scientific computing applications.

**F. Alex Feltus** is a professor in computational biology in the Department of Genetics and Biochemistry at Clemson University. His group studies biomedical, agricultural and cyberinfrastructure problems.

**Stephen P. Ficklin** is an associate professor in computational biology and bioinformatics at the Department of Horticulture at WSU and is focused on the development of computational tools, infrastructure and data models for the identification of biomarkers of agricultural importance phenotypes.
